# Regulation of p53 by *Helicobacter pylori*

**DOI:** 10.18632/oncotarget.698

**Published:** 2012-10-08

**Authors:** Alexander Zaika, Jinxiong Wei, Jennifer Noto, Richard M. Peek

**Affiliations:** Department of Surgery, Department of Cancer Biology, Vanderbilt University Medical Center, Nashville, TN; Department of Surgery, Vanderbilt University Medical Center, Nashville, TN; Division of Gastroenterology, Hepatology & Nutrition, Vanderbilt University Medical Center, Nashville, TN; Division of Gastroenterology, Hepatology & Nutrition, Vanderbilt University Medical Center, Nashville, TN

Bacterial pathogen *Helicobacter pylori* colonizes the stomachs of approximately half of the world's population causing gastritis, peptic ulceration, and gastric MALT lymphoma. This bacterium is also the strongest identified risk factor for the development of distal gastric cancer [1]. Bacterial virulence and host factors each play a role in determining the progression to cancer. Virulence factors allow *H. pylori* to persist in the stomach for decades producing a chronic inflammatory response. DNA damage induced as a result of inflammation is potentially mutagenic and contributes to gastric tumorigenesis. *H. pylori* can also directly compromise the genomic integrity of host cells and induce DNA double-strand breaks [2]. *H. pylori* strains, harboring the *cag* pathogenicity island (*cag* PAI), a 40-kb DNA fragment that encodes a type IV secretion system (T4SS), are associated with an increased risk of cancer. The T4SS forms a syringe-like pilus structure used to inject bacterial components directly into host cells. This triggers aberrant activation of multiple oncogenic pathways and can modify the host immune response [1].

One of the critical host factors that counteracts tumorigenesis is the p53 tumor suppressor, which controls cellular responses to DNA damage and oncogenic stress. Recent studies suggest, however, that p53 functions in an isoform-specific manner. The human Δ133p53 isoform, which is produced from an alternative intragenic promoter, has been suggested to promote tumorigenesis [3]. Indeed, spontaneous tumorigenesis and reduced apoptosis have been reported in mice expressing the Δ122p53 protein that mimics the human Δ133p53 isoform [4]. In human colon adenomas, Δ133p53 protein has been found to inhibit p53-mediated replicative senescence and an increase of Δ133p53 expression may signal an escape from the senescence barrier during the progression from adenoma to carcinoma [5]. Elevated levels of Δ133p53 have also been reported in a number of other human tumors.

In a recent publication, we described how *H. pylori* affects the expression of p53 and provided evidence that this pathogen alters the expression profile of p53 protein isoforms [6]. We found that interaction of *H. pylori* with gastric epithelial cells, mediated via the *cag* PAI, induces Δ133p53 and Δ160p53 isoforms in human cells. These isoforms inhibit the transcriptional activity of p53 and promote survival of epithelial cells infected with *H. pylori*. At a molecular level, *H. pylori* infection increases protein-protein interactions between p53 and Δ133p53 isoforms that hamper p53-dependent transcription. Δ133p53 also inhibits the transcriptional activity of another member of the p53 protein family, TAp73, which has been shown to mediate *H. pylori*-induced apoptosis [7]. The induction of the truncated p53 isoform was found not only in human cells but also in *H. pylori*-infected Mongolian gerbils, a model in which pre-malignant lesions and gastric cancer are similar to those observed in humans. This orthologous isoform of p53 was identified as gerbil Δ153p53 protein.

Our studies have also shown that p53 isoforms are engaged in regulation of the NF-κB pathway. Increased expression of Δ133p53 was found to lead to the induction of NF-κB transcriptional activity and NF-κB target genes, such as pro-inflammatory cytokines and anti-apoptotic Bcl-2 family proteins. Δ133p53, therefore, inhibits apoptosis not only by directly inhibiting p53 and p73 but also by promoting pro-survival signals mediated by the NF-κB pathway. Mechanistically, activation of NF-κB by Δ133p53 is linked to inhibition of p53, given that NF-κB activation is hampered in p53-null cells. It is possible that these mechanisms of NF-κB regulation by p53 isoforms are not unique for *H. pylori* infection and may be employed by other pathogens.

The regulation of p53 isoforms by *H. pylori* is complex and involves multiple mechanisms. We found that increased phosphorylation of the c-Jun protein and activation of AP-1-dependent transcription are important events for transcriptional up-regulation of p53 isoforms. The corresponding binding site for AP-1 was identified in the promoter region that controls expression of p53 isoforms. Notably, AP-1 activation requires a functional *cag*-PAI. Strains lacking the T4SS were found to be deficient in activation of p53 isoforms. Our studies also suggested that non-transcriptional mechanisms contribute to increased expression of dominant-negative p53 isoforms.

In an earlier investigation, we reported that *H. pylori* accelerates ubiquitination and proteasomal degradation of p53 in gastric epithelial cells [8]. Degradation of p53 and inhibition of its functional activity by *H. pylori* has also been found to occur in B-cells, the cell of origin of MALT lymphoma [9]. *H. pylori*-induced degradation of p53 is mediated by cellular HDM2 protein and bacterial protein CagA, which is delivered by the T4SS into epithelial cells. Interestingly, induction of p53 isoforms is independent of CagA but requires the functional T4SS, implicating additional bacterial factors into the regulation of p53 isoforms. Further investigation is needed to elucidate these mechanisms.

In summary, our studies suggest that in the process of evolutionary adaptation to the host environment, *H. pylori* has developed clever mechanisms to counteract the function of p53. Inhibition of p53 may provide advantages to *H. pylori* and allow this organism to alter cellular homeostasis without triggering cell cycle arrest or apoptosis. As a consequence, however, such alteration may increase the risk of tumor development. Further studies of p53 regulation will contribute to a deeper understanding of tumorigenesis associated with *H. pylori* infection.
